# Relationship between paraspinal muscle properties and bone mineral density based on QCT in patients with lumbar disc herniation

**DOI:** 10.1186/s12891-024-07484-0

**Published:** 2024-05-07

**Authors:** Ze Li, Junrong Chen, Jian Yang, Ran Wang, Wenbin Wang

**Affiliations:** 1 School of Sports Medicine and Health, Chengdu Sport University, No.2, Tiyuan Road, Chengdu, Sichuan China; 2 Department of radiology, Sichuan Province Orthopedic Hospital, No.132, West Section of 1st Ring Road, Chengdu, Sichuan China

**Keywords:** Volumetric bone mineral density, Paraspinal muscle, The proton density fat fraction, Muscle cross-sectional area, Lumbar disc herniation

## Abstract

**Objective:**

Increasing research suggests that paraspinal muscle fat infiltration may be a potential biological marker for the assessment of osteoporosis. Our aim was to investigate the relationship between lumbar paraspinal muscle properties on MRI and volumetric bone mineral density (vBMD) based on QCT in patients with lumbar disc herniation (LDH).

**Methods:**

A total of 383 patients (aged 24–76 years, 193 females) with clinically and radiologically diagnosed LDH were enrolled in this retrospective study. The muscle cross-sectional area (CSA) and the proton density fat fraction (PDFF) were measured for the multifidus (MF), erector spinae (ES) and psoas major (PS) at the central level of L3/4, L4/5 and L5/S1 on lumbar MRI. QCT was used to measure the vBMD of two vertebral bodies at L1 and L2 levels. Patients were divided into three groups based on their vBMD values: normal bone density group (> 120 mg/cm^3^), osteopenia group (80 to 120 mg/cm^3^) and osteoporosis group (< 80 mg/cm^3^). The differences in paraspinal muscle properties among three vBMD groups were tested by one-way ANOVA with post hoc analysis. The relationships between paraspinal muscle properties and vBMD were analyzed using Pearson correlation coefficients. Furthermore, the association between vBMD and paraspinal muscle properties was further evaluated using multiple linear regression analysis, with age and sex also included as predictors.

**Results:**

Among the 383 LDH patients, 191 had normal bone density, 129 had osteopenia and 63 had osteoporosis. In LDH patients, compared to normal and osteopenia group, paraspinal muscle PDFF was significantly greater in osteoporosis group, while paraspinal muscle CSA was lower (*p* < 0.001). After adjusting for age and sex, it was found that MF PDFF and PS CSA were found to be independent factors influencing vBMD (*p* < 0.05).

**Conclusion:**

In patients with LDH, paraspinal muscle properties measured by IDEAL-IQ sequence and lumbar MR scan were found to be related to vBMD. There was a correlation between the degree of paraspinal muscle PDFF and decreasing vBMD, as well as a decrease paraspinal muscle CSA with decreasing vBMD. These findings suggest that clinical management should consider offering tailored treatment options for patients with LDH based on these associations.

## Background

Lumbar disc herniation (LDH) is a common spinal disorder that affects a significant portion of the population, causing pain, disability, and decreased quality of life [1]. Previous studies have reported that patients with LDH often exhibit alterations in paraspinal muscle properties, such as muscle atrophy, fat infiltration, and reduced muscle strength [2, 3]. These paraspinal muscle degenerations may be closely related to the development and progression of LDH [4].

The paraspinal muscles are crucial for maintaining spinal stability and providing support for the spine [5]. They play a significant role in sustaining proper posture, facilitating movement, protecting the spinal structures, and distributing loads throughout the lumbar spine [6]. It is widely accepted that degeneration of paraspinal muscles is associated with multiple spinal degenerative features [7]. Moreover, considering that muscles and bones are functional units with synchronicity and interactions at both the mechanical and biological levels [8], assessing the relationship between paraspinal muscle properties and the vertebral column holds increasing importance. Muscle atrophy and muscle fatty infiltration have been identified as properties changes that occur in muscle degeneration [9].

The assessment of the paraspinal muscle and vertebrae using various imaging methods can offer valuable insights into the pathophysiological mechanisms of the musculoskeletal system. Magnetic resonance imaging (MRI) is considered a good method for the quantifying skeletal muscles, as it provides detailed images that distinguishing between fat and non-fat components [10]. The chemical shift-encoded iterative decomposition of water and fat with echo asymmetry and least squares estimation (IDEAL-IQ) sequence, as a new quantitative MR technique, is emerging as a preferred method for noninvasively quantifying the proton density fat fraction (PDFF) in tissue [11]. Quantitative computed tomography (QCT) can address the limitations of dual-energy X-ray absorptiometry (DXA) [12] by assessing the true volumetric bone mineral density (vBMD) per square centimeter, providing improved specificity and sensitivity [13].

Several studies have investigated the relationship between the properties of paraspinal muscles and vBMD in healthy populations [14, 15] or patients with low back pain [16]. These studies demonstrated that paraspinal muscle fat infiltration was greater in patients with osteoporosis than in patients with normal or osteopenia. Paraspinal muscle fat infiltration may be a biological marker of lumbar BMD [17]. However, to the best of our knowledge, no study has specifically explored this relationship in patients with LDH. Investigating the link between paraspinal muscle size, intramuscular fat infiltration and vertebral bone mineral density is essential for optimizing LDH treatment strategies and improving patient prognosis.

Therefore, the purpose of this study was to investigate the relationship between paraspinal muscle properties measured by the IDEAL-IQ MRI sequence and vBMD measured by QCT in patients with LDH.

## Methods

### Study population

This retrospective study was approved by the Institutional Review Board and the Ethics Committee of Sichuan Province Orthopedic Hospital, without the requirement to obtain informed consent from patients (KKY2021-028-01).

We reviewed patients who underwent both 3.0T MRI and QCT scans between April 2022 and August 2023. Patients diagnosed with LDH based on a combination of clinical history, physical examination, and imaging assessments at our hospital were selected. The QCT examination was an opportunistic QCT within a month’s time of receiving the MRI scan and therefore did not add additional radiation. All patients were outpatients and inpatients who received only conservative treatments including medications, acupuncture, and functional exercises, and all of them showed significant improvement in their symptoms after a period of conservative treatment.

**Fig. 1:**
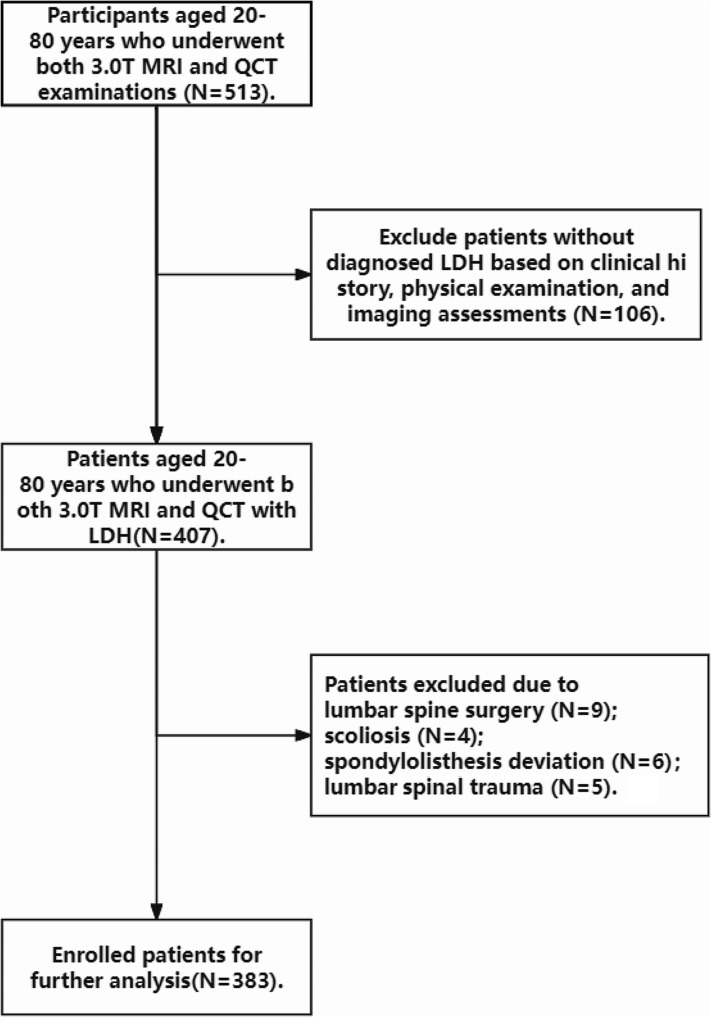
Exclusion process for inclusion of this study population

The inclusion criteria were as follows: [1] aged between 20 and 80 years and [2] had no other chronic or serious organic diseases. The exclusion criteria were as follows: [1] history of lumbar spine surgery; [2] presence of other diseases that may affect skeletal muscles, such as spinal trauma and tumors; and [3] presence of congenital anomalies that affect musculoskeletal relationships, such as scoliosis and spondylolisthesis deviation. Finally, a total of 383 LDH patients (aged 24–76 years, 193 females) were enrolled in this study (Fig. 1). The basic characteristics (age, sex, body mass index (BMI)) of all patients were collected. Table 1 shows the baseline characteristics of the study population.

### MRI examination

Patients were scanned on a 3.0T MR system (SIGNA Architect, GE Healthcare, Milwaukee, USA). Routine lumbar MRI and axial IDEAL-IQ sequence scans were performed for each patient. The imaging protocol included the following: an axial IDEAL-IQ sequence with repetition time (TR) 8.0 ms, effective echo time (TE) 3.6 ms, slice thickness 1.0 mm, bandwidth 62.50 Hz, field of view 256 × 256 mm, voxel size 2.0 × 2.0 × 2.0 mm, auto flip angle 4°; and acquisition time 182 s; Six echoes were used for the quantification of PDFF; an axial T2-weighted spin-echo sequence with TR 3684 ms and TE 123 ms, slice thickness of 3 mm, bandwidth 50.00 Hz, field of view of 200 × 200 mm, voxel size 0.5 × 0.8 × 3.0 mm, and a total of 14 scanning layers.

### QCT examination

Spiral CT imaging of the L1 and L2 vertebral bodies was performed on a CT scanner (SIEMENS SOMATOM go. up and go. fit, Germany) with the QCT Pro analysis software (Mindways Software, Inc. Austin, Texas, USA). The acquisition parameters were as follows: 120 kV, 214 mAs, 1.0 mm reconstructed slice thickness, and 500 mm field of view. The scanning range was from the superior margin level of the T12 vertebral body to the inferior margin level of the L3 vertebral body.

### Data processing

**Fig. 2:**
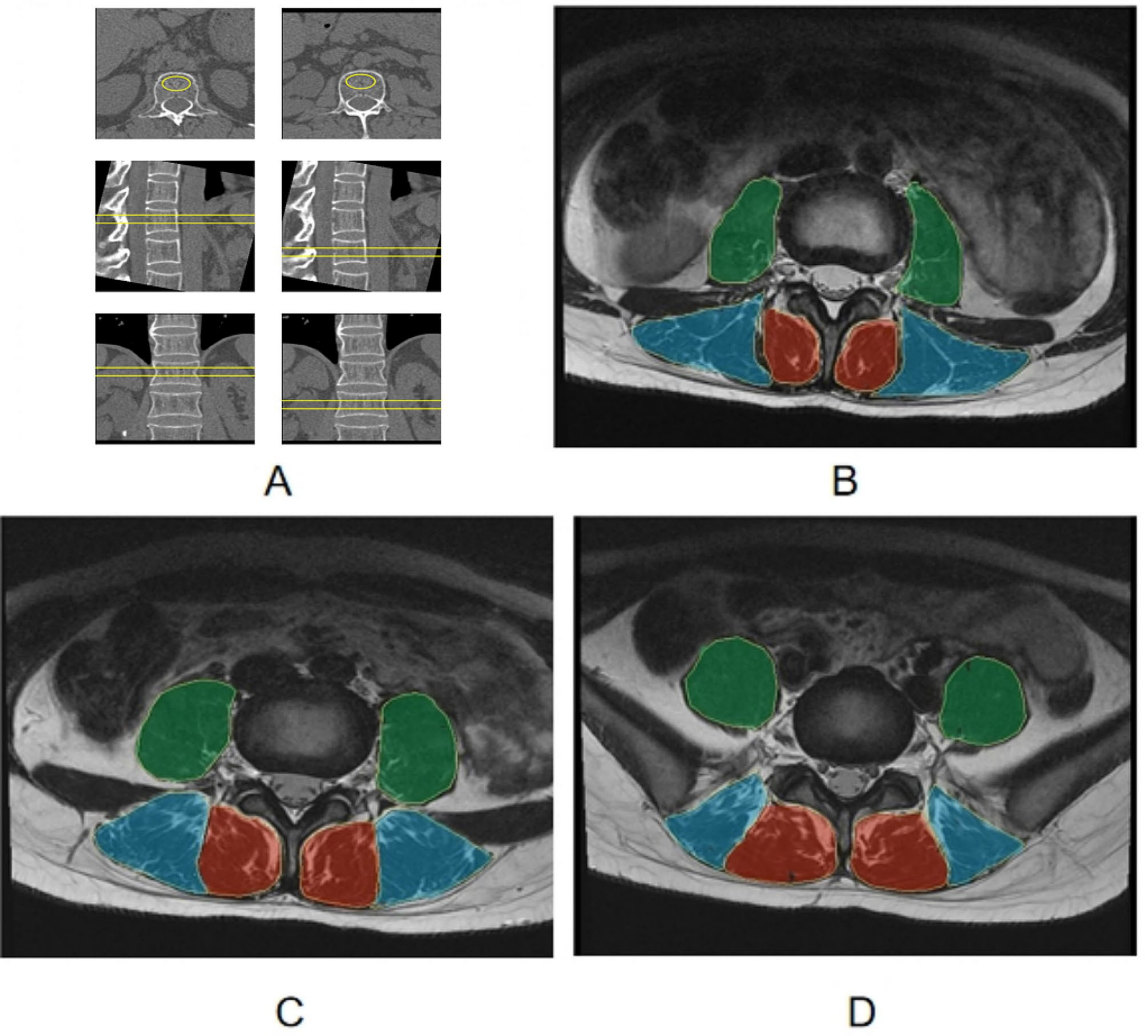
ROI delineation for measuring the vBMD and paraspinal muscle PDFF and CSA. ROIs for vBMD measurements in L1 and L2 vertebral bodies were drawn automatically on the QCT pro workstation in three-plane images with manual adjustment to avoid the cortical bones **(A)**. T2-weighted images were utilized to delineate ROIs for measuring the CSA and PDFF value. ROIs for the bilateral paraspinal muscles (MF in red, ES in blue, and PS in green) were manually delineated at the central level of L3/4 **(B)**, L4/5 **(C)**, and L5/S1 **(D)** on axial T2-weighted images. Subsequently, the same ROIs were automatically copied to the IDEAL-IQ images and then simply adjusted to measure PDFF values. vBMD, volumetric bone mineral density; PDFF, the proton density fat fraction; CSA, cross-sectional area; QCT, quantitative computed tomography; ROI, region of interest; MF, multifidus; ES, erector spinae; PS, psoas major

All vBMD measurements were conducted by one professionally trained radiologist with more than two years of experience who was blinded to the muscle measurements. The measurements were repeated after three months, with 30 images randomly selected by this measurer to obtain intra-observer agreement. The QCT image files were transferred to the QCT pro workstation with a bone densitometry analysis software (Mindways Software, Inc.). As illustrated in Fig. 2A, the vBMD of L1 and L2 vertebral bodies was respectively measured by placing a 3D region of interest (ROI) at the center of the vertebral body, while avoiding the cortical bone and basivertebral plexus travel areas. Subsequently, the software automatically derived the average vBMD value within each ROI. Finally, the mean vBMD of the two vertebral bodies was computed and utilized for later analysis. All patients were divided into the normal bone density group (vBMD > 120 mg/cm^3^), the osteopenia group (vBMD 80 to 120 mg/cm^3^), and the osteoporosis group (vBMD < 80 mg/cm^3^) following the guidelines recommended by the International Society for Clinical Densitometry (ISCD) in 2007 [18].

All measurements of paraspinal muscle properties were performed by another independent radiologist with more than two years of experience who was unaware of the vBMD measurements. Three months later, the radiologist performed a repeat quantitative analysis of 30 randomly selected images after the first measurement to assess intra-observer reliability. As depicted in Fig. 2B and D, the CSA and PDFF values of the bilateral paraspinal muscles, including the multifidus (MF), erector spinae (ES) and psoas major (PS), were obtained on a region of interest (ROI) basis at the center of the intervertebral disc of L3/4, L4/5, and L5/S1, respectively. The radiologist manually delineated these ROIs, including muscle, intramuscular fat, and soft tissue, along the edges of the paraspinal muscles on axial T2-weighted images to obtain the paraspinal muscles CSA. The IDEAL-IQ images were processed on the Workstation (Advantage Windows 4.7, GE Healthcare, USA) to calculate the PDFF maps, which were subsequently co-registered to axial T2-weighted images. The same ROIs were then automatically copied to the IDEAL-IQ images, and simply adjusted to measure PDFF values. The mean CSA and PDFF of L3/4, L4/5, and L5/S1 were calculated to represent the overall muscle profiles in the lower lumbar region. It took approximately 15 min to draw all the paraspinal muscle ROIs at all three levels for each subject.

### Statistical analysis

All data were analyzed using IBM SPSS Statistics for Windows, Version 22.0 (IBM Corp, Armonk, NY, USA). The normality of the data was analyzed using the P-P Chart. Based on the data distribution characteristics, the mean ± standard deviation (SD) was used to describe the data. Differences in paraspinal muscle properties, age, and BMI among the three vBMD groups were tested by one-way ANOVA with post hoc analysis (LSD). Independent samples t-tests were utilized to examine differences in paraspinal muscle properties, age, and BMI between the male and female groups. The correlations between vBMD and paraspinal muscle properties, age, BMI were analyzed using Pearson correlation coefficients. Additionally, the relationship between vBMD and the paraspinal muscle properties was further tested using multiple linear regression analysis, with age and sex also included as predictors. *p* < 0.05 indicated statistical significance.

Intra-observer precision was evaluated from measurements of 30 randomly selected MR images performed twice by a single radiologist with time a 3-month time interval. Intra-observer reproducibility was determined using the intraclass correlation coefficient (ICC).

## Results

### Repeatability evaluation of CSA and PDFF value

The assessment revealed good agreement between the first and second measurements, demonstrating the reliability of the measurements. The ICCs for Intra-observer reproducibility used for the paraspinal muscle CSA and PDFF were both > 0.8, with ICCs ranging from 0.833 to 0.966 (Table 1).

**Table 1:** The intra-observer reproducibility values for the paraspinal muscle CSA and PDFF measurements

		ICC (95% CI)	*p* values
CSA (mm^2^)	MF	0.885(0.772–0.943)	< 0.001**
	ES	0.833(0.677–0.917)	< 0.001**
	PS	0.966(0.929–0.983)	< 0.001**
PDFF (%)	MF	0.927(0.793–0.970)	< 0.001**
	ES	0.866(0.664–0.942)	< 0.001**
	PS	0.916(0.830–0.959)	< 0.001**

The double asterisks (**) indicate *p* < 0.01. ICC, intraclass correlation coefficient; 95% CI, 95% confdence interval; CSA, cross-sectional area; PDFF, the proton density fat fraction; MF, multifidus; ES, erector spinae; PS, psoas major

### Baseline characteristics of the patients

A total of 383 patients aged 24–76 years were enrolled in this study, including 190 males and 193 females with LDH. The mean age of the patients was 51.69 ± 11.06 years, and the distributions of the patients according to QCT criteria were as follows: normal bone density (*n* = 191), osteopenia (*n* = 129), and osteoporosis groups (*n* = 63). The clinical characteristics, including paraspinal muscle properties of the patients in the normal, osteopenia and osteoporosis groups are presented in Table 2.

### Comparison of clinical characteristics and paraspinal muscle properties among the normal, osteopenia and osteoporosis groups

**Fig. 3:**
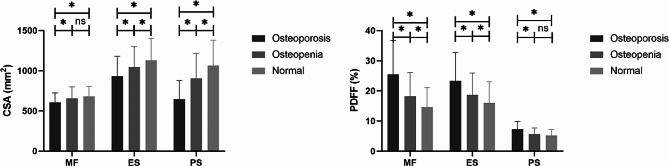
Histograms of paraspinal muscle properties among the normal bone density, osteopenia, and osteoporosis groups. The *p* values are from post hoc analysis. The single asterisk (*) and double asterisks (**) indicate *p* < 0.05 and *p* < 0.01, respectively. MF, multifidus; ES, erector spinae; PS, psoas major; CSA, cross-sectional area; PDFF, the proton density fat fraction

Table 2 summarizes the demographic characteristics of patients and paraspinal muscle measurements. Significant differences in age were found between any two of the three groups (all *p* < 0.05), while no significant difference in BMI was found among the three groups (*p* = 0.172). It was found that the paraspinal muscle CSA and PDFF showed statistically significant differences among the three groups (*p* < 0.001). As displayed in Table 2; Fig. 3, compared with those in osteopenia group and normal bone density group, osteoporosis group exhibited smaller paraspinal muscle CSA (all *p* < 0.05); and higher paraspinal muscle PDFF (all *p* < 0.05). Between osteopenia group and normal bone density group, no significant difference was observed between the MF CSA (*p* = 0.075) and PS FF (*p* = 0.055).

**Table 2:** The clinical characteristics and paraspinal muscle properties of the patients in the normal bone density, osteopenia, and osteoporosis groups

		Normal (*N* = 191) Mean ± SD	Osteopenia (*N* = 129) Mean ± SD	Osteoporosis (*N* = 63) Mean ± SD	All (*N* = 383) Mean ± SD	*p* values
Sex	Male/ Female	*N* = 113/78	*N* = 64/65	*N* = 13/50	*N* = 190/193	
Age (years)		45.37 ± 9.89	55.40 ± 7.96	63.25 ± 5.93	51.69 ± 11.06	< 0.001**
BMI (kg/m^2^)		24.56 ± 2.72	24.51 ± 3.36	25.37 ± 4.18	24.68 ± 3.23	0.172
vBMD (mg/cm^3^)		154.46 ± 21.43	101.98 ± 11.39	65.06 ± 13.07	122.08 ± 38.69	< 0.001**
CSA (mm^2^)	MF	682.49 ± 121.58	656.40 ± 142.93	607.01 ± 116.38	661.29 ± 130.82	< 0.001**
	ES	1131.03 ± 271.63	1046.78 ± 253.80	932.20 ± 246.78	1069.95 ± 270.82	< 0.001**
	PS	1064.89 ± 314.91	904.97 ± 309.13	646.12 ± 234.48	942.14 ± 335.74	< 0.001**
PDFF (%)	MF	14.66 ± 6.45	18.20 ± 7.87	25.44 ± 11.23	17.63 ± 8.74	< 0.001**
	ES	16.02 ± 7.02	18.69 ± 7.27	23.36 ± 9.34	18.13 ± 7.95	< 0.001**
	PS	5.25 ± 1.98	5.71 ± 2.00	7.30 ± 2.61	5.74 ± 2.21	< 0.001**

The *p* values are from one-way ANOVA. The double asterisks (**) indicate *p* < 0.01. BMI, body mass index; vBMD, volumetric bone mineral density; MF, multifidus; ES, erector spinae; PS, psoas major; CSA, cross-sectional area; PDFF, the proton density fat fraction; SD, standard deviation

### Comparison of clinical characteristics and paraspinal muscle properties between groups of different gender

Table 3 outlines the differences in paraspinal muscle properties and demographic characteristics between male and female groups. Age, vBMD, and paraspinal muscle properties showed significant differences between the two groups (*p* < 0.001). The paraspinal muscle CSA was larger in males compared to females (all *p* < 0.001). Except for PS PDFF, males had lower paraspinal muscle PDFF than females (all *p* < 0.001). However, no significant difference in BMI was observed between the sexes (*p* = 0.204).

**Table 3:** The clinical characteristics and paraspinal muscle properties between the male and female groups

		Male (*N* = 190) Mean ± SD	Female (*N* = 193) Mean ± SD	*p* values
Age (years)		48.98 ± 10.88	54.35 ± 10.60	< 0.001**
BMI (kg/m^2^)		24.88 ± 2.94	24.46 ± 3.47	0.204
vBMD (mg/cm^3^)		131.41 ± 34.15	112.89 ± 40.72	< 0.001**
CSA (mm^2^)	MF	717.39 ± 128.16	606.05 ± 108.17	< 0.001**
	ES	1194.19 ± 262.23	947.64 ± 218.91	< 0.001**
	PS	1191.19 ± 258.29	696.97 ± 191.82	< 0.001**
PDFF (%)	MF	13.05 ± 5.63	22.13 ± 8.93	< 0.001**
	ES	14.74 ± 6.42	21.46 ± 7.91	< 0.001**
	PS	5.55 ± 2.17	5.93 ± 2.25	0.093

The *p* values are from independent samples t-test. The double asterisks (**) indicate *p* < 0.01. BMI, body mass index; vBMD, volumetric bone mineral density; MF, multifidus; ES, erector spinae; PS, psoas major; CSA, cross-sectional area; PDFF, the proton density fat fraction; SD, standard deviation

### Correlations between vBMD and age, BMI, and paraspinal muscle properties

Table 4 presents the Pearson correlation coefficients between vBMD and age, BMI, and paraspinal muscle properties. Age showed a strongly negatively correlation with vBMD (*r*= -0.694, *p* < 0.01). Pearson’s correlation analysis revealed that vBMD was mildly to moderately positively correlated with the MF CSA (*r* = 0.202, *p* < 0.01), the ES CSA (*r* = 0.278, *p* < 0.01), the PS CSA (*r* = 0.460, *p* < 0.01). The vBMD was mildly to moderately negatively correlated with the MF PDFF (*r*= -0.484, *p* < 0.01), the ES PDFF (*r*= -0.359, *p* < 0.01), the PS PDFF (*r*= -0.289, *p* < 0.01). However, there was no significant correlation between vBMD and BMI (*p* > 0.05).

**Table 4:** Pearson correlation coefficients between the paraspinal muscle properties, age and BMI versus vBMD.

		*r* values	*p* values
Age (years)		-0.694**	< 0.01**
BMI (kg/m^2^)		-0.063	0.222
CSA (mm^2^)	MF	0.202**	< 0.01**
	ES	0.278**	< 0.01**
	PS	0.460**	< 0.01**
PDFF (%)	MF	-0.484**	< 0.01**
	ES	-0.359**	< 0.01**
	PS	-0.289**	< 0.01**

The double asterisks (**) indicate *p* < 0.01. CSA, cross-sectional area; PDFF, the proton density fat fraction; vBMD, volumetric bone mineral density; BMI, body mass index. MF, multifidus; ES, erector spinae; PS, psoas major

### Independent predictors of vBMD according to the multiple linear regression model

As shown in Table 5, the relationship between vBMD and paraspinal muscle properties was further tested using multiple linear regression analysis, with age and sex also included as predictors. The R^2^ and adjusted R^2^ of the overall linear model were 0.732 and 0.536, respectively (*p* < 0.05). The model revealed that sex (β = 12.151, *p* = 0.006), age (β = -1.842, *p* < 0.001), the PS CSA (β = -0.030, *p* < 0.001) and the MF PDFF (β = -0.699, *p* = 0.010) were significant and independent factors of vBMD in patients with LDH. However, the MF CSA (*p* = 0.557), the ES CSA (*p* = 0.124), the ES PDFF (*p* = 0.397) and the PS PDFF (*p* = 0.328) were not associated with vBMD.

**Table 5:** Independent predictors of vBMD analyzed by multiple linear regression model

		Unstandardized		Standardized	T	*p* value
		β	SE	β		
Sex		12.151	4.422	0.157	2.748	0.006**
Age (years)		-1.842	0.154	-0.527	-11.950	< 0.001**
CSA (mm^2^)	MF	-0.008	0.013	-0.026	-0.588	0.557
	ES	0.010	0.006	0.069	1.542	0.124
	PS	0.030	0.008	0.258	3.826	< 0.001**
PDFF (%)	MF	-0.699	0.270	-0.158	-2.594	0.010*
	ES	0.242	0.286	0.050	0.848	0.397
	PS	-0.718	0.734	-0.041	-0.979	0.328

The single asterisk (*) and double asterisks (**) indicate *p* < 0.05 and *p* < 0.01, respectively. PDFF, the proton density fat fraction; MF, multifidus; ES, erector spinae; PS, psoas major; SE standard error

## Discussion

The innovation of this study was to use quantitative techniques, including QCT and the IDEAL-IQ sequence, to explore the changes of lumbar spine vBMD and paraspinal muscle properties in patients with LDH. Our findings revealed that in LDH patients, the PDFF of paraspinal muscles increases as vBMD decreases, while the CSA of paraspinal muscles decreases as vBMD decreases. Our results also showed that the PS CSA and MF PDFF are independent influential factors of vBMD. This finding suggests a close relationship between muscle mass, muscle size and bone density, indicating that there is an interconnected and interacting system between muscle and bone.

Muscle and bone interact throughout a person’s life, and there are currently two mechanisms explaining this interaction. One mechanism involves mechanical loads [19], where the tension generated by the muscle stimulates osteogenic activity in the bone, leading to responses from osteoblasts and osteocytes. The other mechanism involves endocrine factors [20], where the muscle produces biochemical signals during exercise, including hormones and growth factors, which impact the coupling process of bone formation and resorption. Muscle CSA has been widely used to estimate muscle volume as an indirect indicator of muscle strength [21]. Muscle fat infiltration is an important manifestation of skeletal muscle aging and reflects the decline in skeletal muscle function and strength [22]. Both muscle CSA and PDFF reflect the mechanical tension between muscle and bone. Muscle fat infiltration or muscle atrophy affects the mechanical stimulation of the corresponding area of bone.

However, it is controversial about the relationship between BMD and muscle size. Yang et al. [15] showed that vertebral BMD was associated with paraspinal muscle fat infiltration but not muscle size. This difference may be attributed to variations in the study population, as we focused on an LDH-prevalent population, potentially leading to distinct findings. Our study demonstrated a negative correlation between vertebral vBMD and the paraspinal muscle PDFF, and a positive correlation between vertebral vBMD and the paraspinal muscle CSA, which aligns with the findings of previous similar studies. For example, Li et al. [23] found that both muscle CSA and PDFF in the muscles near the hip joint correlate with proximal femur BMD. This finding suggests that muscle fat infiltration and muscle atrophy may progress simultaneously with age, collectively impacting vBMD. Our study revealed that degenerating paraspinal muscles may contribute to a decrease in vBMD, and this change that is also present in the LDH patients. These findings suggest that we should pay attention to the management of CSA and PDFF of paraspinal muscles in patients with LDH.

We found that among the paraspinal muscles, the PS CSA and MF PDFF were found to be independent influential factors of vBMD. This may be due to the role of the MF as the primary stabilizing muscle of the lumbar spine [24]. Anatomical studies have shown that the MF has the largest paraspinal cross-sectional area and is located in the innermost portion of the spine, providing substantial stabilizing support to the spine [25]. In addition, the PS is a core muscle group that represents the overall stability of the body, and it originates from the lumbar vertebrae [26]. Both the MF and PS exert varying degrees of tensioning load on the lumbar spine. Changes in lumbar paraspinal muscle properties may be an important indicator of lumbar spine BMD [27], and the presence of low BMD also reflects alterations in paraspinal muscle properties. Understanding the relationship between paraspinal muscle properties and BMD in patients with LDH is crucial for comprehending the issue of low back pain and can help provide clinicians with ideas for conservative treatment options.

In addition, our study revealed significant differences in age, vBMD and paraspinal muscle properties between males and females. Compared with females, males exhibited greater paraspinal muscle CSA and lower paraspinal muscle PDFF values. Furthermore, males had higher vBMD than females, and gender was also an independent factor of vBMD. Some studies have also reported lower BMD and greater muscle fat infiltration in females than in males [28, 29]. This may be because the musculoskeletal relationship can be affected by hormones such as estrogen deficiency [30]. The decline in estrogen levels in menopausal women leads to bone mass loss and an increase in muscle fat infiltration [31], leading to a greater degree of muscle fat infiltration in females compared to males.

The results also revealed a strong negative correlation between vBMD and age in LDH patients, and age was also identified as an independent factor of vBMD, which is consistent with the findings of epidemiological studies in the United States [32]. Aging has a non-negligible impact on muscle degeneration. On the other hand, the osteoporosis group had a slightly higher BMI than the normal bone density group and the osteopenia group, but there was no statistically significant difference in BMI among the three bone mass groups. This is contrary to findings of Han et al. [33], which reported a statistically significant difference in BMI among the three bone mass groups, with the lowest BMI observed in the osteoporosis group. Low BMI is always recognized as a risk factor for osteoporosis [34]. However, the study by Zhu et al. [35] demonstrated that the positive correlation between BMI and BMD was attenuated at high BMI, and that fat mass by DXA were significant predictors of BMD measurement. Therefore, the results of this study suggest that the degree of paraspinal muscle fat infiltration (e.g., intramuscular and intermuscular fat in localized muscles) may not be affected by overall body fat.

Low back pain is a common clinical symptom in patients with LDH, and it is often attributed to fatty degeneration of the paraspinal muscles [36]. The duration of symptoms and compression of nerve roots in lumbar disc herniation may have a direct effect on the degeneration of the paraspinal muscles. Stevens et al. [2] showed that unilateral LDH causes increased fat infiltration and atrophy of the ipsilateral multifidus muscle. In patients with LDH with long-term chronic radicular compression, the level of disc herniation is associated with altered paraspinal muscle morphology at or below the same or lower pathologic level [37]. Similarly, Kjaer et al. [38] demonstrated a strong correlation between low back pain and paraspinal muscle fatty infiltration in adults.

However, the effect of symptom duration and severity on paraspinal muscle properties in patients with LDH is unclear. Fortin et al. [39] observed a greater fat component in the paraspinal muscles at the herniation level in patients with symptomatic disc herniation, but there was no significant difference in muscle size. There was no correlation between the visual analogue scale (VAS) score and the paraspinal muscle PDFF in patients with low back pain in a study by Li et al. [40]. Similarly, Danneels et al. [41] reported no difference in the paraspinal muscle CSA between low back pain patients and healthy control subjects. Kilic et al. [42] showed that patients with LDH with different degrees of prominence did not differ in pain level or CSA of the multifidus muscle. Previous studies in the LDH population have focused primarily on the association between disc pathology and paraspinal muscles, with limited exploration of the association between muscle and bone. Our study extends the research on LDH by investigating the musculoskeletal relationship, specifically examining the correlation between BMD and paraspinal muscle properties in the LDH population, rather than being limited to the healthy population.

This study has several limitations. Firstly, this was a cross-sectional observational study, making it challenging to establish a causal relationship between vBMD and paraspinal muscle properties, necessitating long-term follow-up studies. Secondly, the study had an uneven distribution across bone mineral density groups, especially in the osteoporosis group of patients. However, the sample size of patients included in this study was large enough, with a wide age range and a balanced male-to-female ratio, potentially providing insights into bone and muscle development stages throughout life. Thirdly, the study did not account for the dimensions or severity of the patients’ LDH, nor did it consider the duration of the disease or the individuals’ level of mobility. In future studies, we can explore the effect of symptoms and severity on the relationship between paraspinal muscles and BMD in patients with LDH by grouping them into different pain levels or lumbar disc herniation levels.

In future studies, it may be feasible to validate muscle properties as predictors of osteoporosis. It is possible to identify patients with osteoporosis by simply including an MRI scan sequence, eliminating the need for additional radiation from CT scans. Enhancing screening efficiency and reducing costs could alleviate the societal burden. Additionally, analyzing the correlation between muscle and bone in LDH patients is crucial for comprehending LDH and determining appropriate conservative treatments to identify and prevent the development of osteoporosis and its complications.

## Conclusions

In conclusion, this study revealed that in LDH patients, lumbar paraspinal muscle PDFF increases with decreasing vBMD, while paraspinal muscle CSA decreases with decreasing vBMD. The PS CSA and MF PDFF were found to be independent influential factors of vBMD. The correlation between paraspinal muscle properties and vBMD may also be influenced by the anatomical function of different paraspinal muscle groups.

## Data Availability

The datasets used and/or analyzed during the current study are available from the corresponding author on reasonable request.

## Abbreviations

BMI: Body mass index
CSA: Cross-sectional area
DXA: Dual-energy X-ray absorptiometry
ES: Erector spinae
ICC: Intraclass correlation coefficient
IDEAL-IQ: Chemical shift-encoded iterative decomposition of water and fat with echo asymmetry and least squares estimation
LDH: Lumbar disc herniation
MF: Multifidus
MRI: Magnetic resonance imaging
PDFF: Proton density fat fraction
PS: Psoas major
QCT: Quantitative computed tomography
ROI: Region of interest
SD: Standard deviation
SE: Standard error
vBMD: Volumetric bone mineral density.Lumbar disc herniation
